# Pharmacologic Activities of Plant-Derived Natural Products on Respiratory Diseases and Inflammations

**DOI:** 10.1155/2021/1636816

**Published:** 2021-10-04

**Authors:** Deepak Timalsina, Krishna Prasad Pokhrel, Deepti Bhusal

**Affiliations:** Central Department of Chemistry, Tribhuvan University, Kirtipur, Kathmandu 44618, Nepal

## Abstract

Respiratory inflammation is caused by an air-mediated disease induced by polluted air, smoke, bacteria, and viruses. The COVID-19 pandemic is also a kind of respiratory disease, induced by a virus causing a serious effect on the lungs, bronchioles, and pharynges that results in oxygen deficiency. Extensive research has been conducted to find out the potent natural products that help to prevent, treat, and manage respiratory diseases. Traditionally, wider floras were reported to be used, such as *Morus alba*, *Artemisia indica*, *Azadirachta indica*, *Calotropis gigantea*, but only some of the potent compounds from some of the plants have been scientifically validated. Plant-derived natural products such as colchicine, zingerone, forsythiaside A, mangiferin, glycyrrhizin, curcumin, and many other compounds are found to have a promising effect on treating and managing respiratory inflammation. In this review, current clinically approved drugs along with the efficacy and side effects have been studied. The study also focuses on the traditional uses of medicinal plants on reducing respiratory complications and their bioactive phytoconstituents. The pharmacological evidence of lowering respiratory complications by plant-derived natural products has been critically studied with detailed mechanism and action. However, the scientific validation of such compounds requires clinical study and evidence on animal and human models to replace modern commercial medicine.

## 1. Introduction

Respiratory inflammatory disorders comprise several air-mediated diseases such as chronic bronchitis, pulmonary diseases, and asthma. Chronic obstructive pulmonary disease (COPD) is a lung inflammatory disease that is the 5^th^ leading cause of death worldwide [1]. Respiratory inflammation is mainly caused by airway disease, characterized by several complications such as coughing, sneezing, and shortness of breath [2]. The disease can act on both upper and lower airways and worsens the other diseases including rhinosinusitis and tightness of the chest [3]. There are multiple problems associated with respiratory inflammation. The upper inflammation is associated with the common cold, pharyngitis, sinusitis, laryngotracheitis, and epiglottitis, and lower inflammation is associated with bronchiolitis, bronchitis, and pneumonia [4]. The inflammation is also induced by a respiratory virus that infects the epithelial lining of the airways and replicates in it [5]. This inflammation normally leads to type 1 inflammation. Inflammation in the healthy airway results in the activation of antiviral state and clearance of viral infection [6, 7], but in chronically inflamed airways, the response against the virus may impair resulting in sustained inflammation [8, 9] and reduced ability of viral clearance [10, 11]. The acute exacerbations may be triggered by several allergens, pollutants, cold and dry air, smoke inhalations, and several pathogenic bacteria in the airways [12]. Asthma is one of the chronic respiratory diseases marked by reversible airway constriction, eosinophil infiltration, increased mucus production, and nonspecific hyperresponsiveness of the airways [13].

There are several treatment methods for reducing complications of respiratory inflammation that include oxygen therapy, steam therapy, draining mucus from the lungs, and taking antihistamines and bronchodilators. Several steroidal and nonsteroidal drugs are used to lessen down inflammation. Inhaled corticosteroids (ICS) in combination with long-acting beta-agonist (LABA) are recommended in many countries. Long-acting bronchodilators such as salmeterol and formoterol can be used in asthma according to the rate of intrinsic activity. Some ultraclass drugs such as *β*-2 agents [14], olodaterol [15], vilanterol [16], carmoterol, PF-610355, LAS100977, and AZD3199 are recommended for therapy against respiratory diseases. Many of the plants such as *Adiantum capillus*-*veneris*, *Aegle marmelos*, *Aerva javanica var. javanica* [17], *Albizia lebbeck*, *Alhagi maurorum*, and *Alhagi maurorum* were used in respiratory disorders by traditional healers and indigenous people [18]. There are many plant-derived compounds of different classes such as alkaloids, flavonoids, glycosides, lignans, polyphenols, and saponins that are studied for their activities against respiratory disease and inflammation. Some compounds like mangiferin, zingerone, glycyrrhizin, piperine, and forsythiaside A are promising and have evidence of positive results in an animal study. Despite the promising effect of plant-derived natural products, the extensive study of clinical evidence and their toxicological aspect is still lacking. Only some of the compounds have been isolated, and a lesser number of experiments have been done in the human model. This review is aimed at collecting and analyzing the traditional approach, reported natural products, and their pharmacological evidence on respiratory diseases and inflammations with sufficient research gaps and recommendations.

## 2. Methodology

The information on respiratory diseases and inflammations had been retrieved from an extensive literature survey. Systematic literature had been searched by using an online database such as Google Scholar, PubMed, SciFinder, ScienceDirect, Mendeley, and Scopus. Literatures were searched in the online database using keywords such as “Respiratory inflammation”, “Ethnomedicine and respiratory diseases”, “Bioactive compounds and respiratory disease”, and “Respiratory drugs”. The cross-referenced articles were also retrieved. Various books, thesis, proceedings, and news articles were secondary sources of information.

## 3. Current Clinical Practice and Approved Drugs

Respiratory inflammatory diseases like asthma and chronic obstructive pulmonary disease (COPD) are usually treated with effective modern medicines of different classes. Nonsteroidal anti-inflammatory drugs (NSAIDs) is a class of drug that has been used efficiently and commonly in the inhibition of the cyclooxygenase enzyme. The past study showed the prescription of triple therapy for the treatment of pulmonary diseases [19] which suggested the use of a long-acting beta-agonist (LABA) and long-acting muscarinic antagonist (LAMA) in combination with inhaled corticosteroid (ICS) [20]. There is a major development in treating COPD and asthma by the ICS-LABA-LAMA therapy. The most common prescriptions nowadays are LABA and ICS discovered by the physician in Europe [21]. The common uses of 22% ICS and 39% bronchodilators are for lower symptoms and 46% ICS and 67% bronchodilators are for greater symptoms. Due to the limited effect of this medication, a trial for triple therapy is tried in every patient [22]. NSAIDs, bronchodilators (*β*_2_-adrenoreceptor (AR) agonists, muscarinic receptor antagonists, and xanthines) [23], and corticosteroids [24] are a highly recommended initial therapy for most patients individually or in combination with one of the other classes [25]. Nonselective COX inhibitors for reducing respiratory inflammation include aspirin, ibuprofen, naproxen, and diclofenac, and selective COX inhibitors include celecoxib, lumiracoxib, etoricoxib, valdecoxib, and rofecoxib [26]. Among different bronchodilators, fast-acting and short-acting albuterol, terbutaline, and fenoterol are efficiently used, yet long-acting agonists salmeterol and formoterol are best for therapy. Some drugs of class ultra-long-acting *β*_2_ agents indacaterol [14], olodaterol [15], vilanterol [16], carmoterol, PF-610355, LAS100977, AZD3199, etc. had been prescribed for achieving one dose daily [27]. The use of a combination of drugs using *β*_2_ long-acting and antimuscarinic controls the transforming growth factor (TGF)-*β*^1^-mediated inflammation in COPD. The novel antimuscarinic agents such as QAT370, glycopyrronium (NVA237), aclidinium, GSK573719, CHF5407, BEA2180BR, TD4208, PF452297, RBx343E48F0, trospium, and dexpirronium are generally used at a high dose for a prolonged duration of action [27]. Anti-inflammatory and bronchodilator action of xanthines such as bamiphylline, enprofylline, isbufylline, and doxophylline is reported to be used in the treatment of asthma and COPD. The safer use of xanthines inhibits the family of phosphodiesterase (PDE3 and 4) enzymes for long-term improvement in lung function [28]. Different NSAIDs like ibuprofen are used in COVID-19 infection, but there is a lack of studies that shows the association between the use of NSAID and COVID-19 severity. Currently, known antiviral agents like lopinavir/ritonavir and remdesivir have a high affinity to the viral enzyme and could inhibit the synthesis of the nitrogenous base resulting in the inhibition of RNA replication through premature termination of the virus [29]. Anti-inflammatory drugs like corticosteroids had a role in the significant reduction of in-hospital mortality by COVID-19 [30]. During this pandemic of COVID-19, several pulmonary complications from this disease were reported such as mucormycosis and pulmonary aspergillosis [31]. These are life-threatening fungal infections and have a role in complicating pulmonary conditions like asthma, bronchiectasis, and COPD. These pulmonary infections are found to attack patients with low immunity. Many researchers and health personnel assumed it was due to the excessive use of corticosteroids. Corticosteroids are used for the treatment of COVID-19 patients which in turn reduces immunity due to which the patients are prone to be infected by mucormycosis and aspergillosis [32]. Losmapimod, p38, a subfamily of mitogen-activated protein kinase (MAPK) inhibitor, is widely studied and used safely as a single IV infusion of 1 to 3 mg doses. There are no severe effects reported except headache, nausea, and fatigue ([33]). Various reports suggested that this can be appropriate in treating COVID-19 patients [34]. The recent trial in the mouse model supported a similar result [35]. Besides this, p38 was able to cause a pathogenic role in asthma and COPD. The adverse factors causing these diseases activate the p38 which in turn amplifies lung inflammation. The clinically trialed anti-interleukins like benralizumab, daclizumab, reslizumab, MEDI-528, mepolizumab, and lebrikizumab showed improvement in patients by decreasing eosinophils and other exacerbations [36]. The clinical trial of benralizumab revealed the effects in reducing eosinophil and improved lung function but with some headache and nausea effects [37]. Number of trials had been conducted for treating upper airway disorders such as allergic rhinitis, nasal polyps, and chronic rhinosinusitis for which several therapeutics such as omalizumab, mepolizumab, dupilumab, a monoclonal antibody targeted toward IgE, an anti-IL-5 agent, anti-IL-4, and IL-3 had been used. The outcomes of the trials were positive [38].

Several other modern drugs have been discovered and synthesized in the laboratory with promising results. However, the success of low-molecular-weight drugs remains low as respiratory inflammation diseases are complex in etiology. The critical target molecule that is directly associated with the disease process has not been found yet. The plant can be the potent source of such medicine as plants have diverse compositions and complex molecular associations. Recently available techniques are effective but associated with several complications such as cost, demand, and availability. Thus, a new kind of efficient and easily available therapeutics should be introduced for developing new kinds of drugs against respiratory inflammation.

## 4. Ethnomedicinal Practice on Treating Respiratory Complications

Several plants were reported to be used for their anti-inflammatory properties that can be used in acute as well as chronic bronchitis. Ethnomedicinally, the number of plants had been reported based on indigenous knowledge of people and the practice of traditional healers. Plants such as *Morus alba* [39], *Dicliptera bupleuroides*, *Adiantum capillus-veneris*, *Trichodesma indicum*, and *Viburnum grandiflorum* were reported to be traditionally used in Pakistan and Korea for treating whooping cough and the common cold. The decoction of leaves of *Dicliptera bupleuroides* was known to apply externally in the throat for managing the cough by the local people of Kashmir of Pakistan [40]. The milky latex and flower paste of *Calotropis gigantea* found in the Terai forest of western Nepal were reported to be taken orally for the management of cough and bronchitis [41]. Some of the reported plants acting against respiratory disorders, based on traditional knowledge and practices, have been listed in Table 1.

## 5. Plant-Derived Compounds on Treating Respiratory Complications

**Figure 1:**
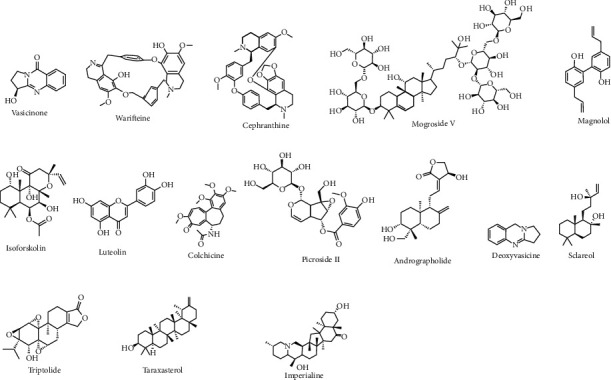
Some major bioactive compounds for respiratory disease.

**Figure 2:**
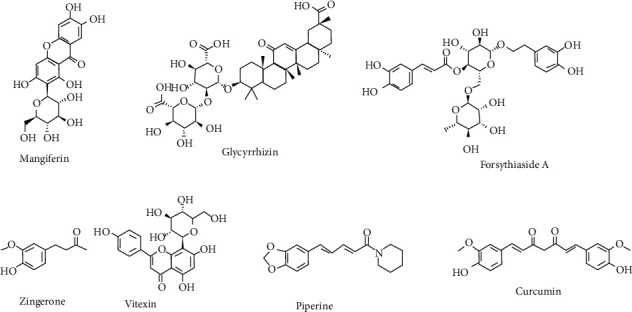
Some promising bioactive compounds for respiratory disease.

The number of compounds (Table 2) derived from plants was reported for the prominent therapeutics against respiratory inflammation. The flavonoids such as kuwanone E, kuwanone G, and norartocarpanone from *Morus alba* [61], sakuranetin from *Baccharis retusa* [62], and pinocembrin (5,7-dihydroxyflavanone) from *Alpinia katsumadai* have been reported to act against respiratory inflammation. The polyphenols such as curcumin (1,7-bis(4-hydroxy-3-methoxyphenyl)-1,6-heptadiene-3,5-dione) from *Curcuma longa* rhizome[63, 64], resveratrol from grapes [65], and luteolin from *Lonicera japonica* [66] were reported to act against respiratory inflammation. The other classes of plant-derived compounds such as alkaloids [67], coumarins [68], and triterpenoids, saponins, and steroids [69–72] were reported to be effective against several kinds of inflammations. Colchicine is a plant alkaloid derivative that could be used as a substitute for commercial colchicine. Colchicine concentrations differ from organ to organ, and colchicine content was demonstrated to be influenced by plant age, seasonality, and location. Colchicine was found to reduce neutrophil elastase concentration in bronchoalveolar lavage fluid in ex-smokers with COPD [73]. Some of the structures of the potent bioactive compounds are given in Figures 1 and 2.

The reported compounds are mostly tested in mice *invivo*, and the inflammation is mainly induced by LPS. The study on the human model and its clinical evidence is still lacking. The possible therapeutics from this promising compound is yet to be studied. The compounds with lower doses and higher activities should be taken into the clinical trial in a sample population.

## 6. Mechanism of Action of Plant-Based Natural Product

**Figure 3:**
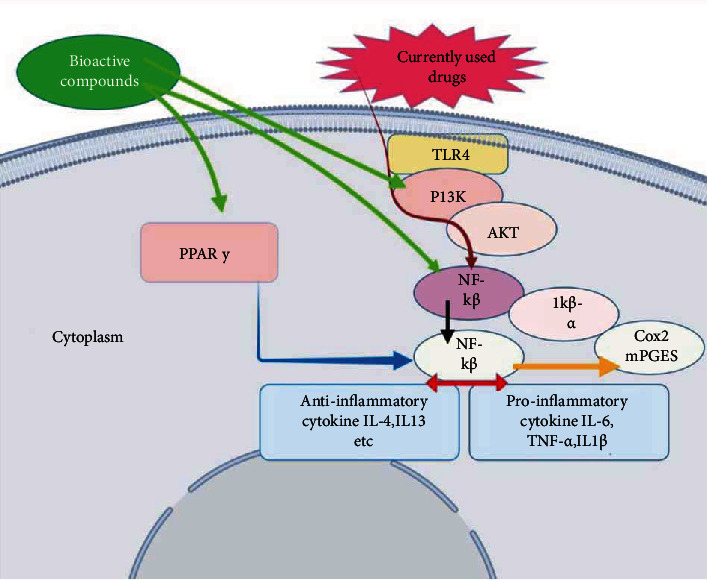
Mechanism of action of a natural product in respiratory inflammation.

The lung inflammation involves the activation of inflammatory cells such as eosinophils, lymphocytes, macrophages, and neutrophils, which serve as the source of different inflammatory mediators such as tumor necrosis factor (TNF-*α*), interleukins (IL-4, IL-1*β*, IL-6, and IL-5), histamine, prostaglandins, nitric oxide, and leukotriene. The release of these inflammatory mediators causes several abnormalities in the lungs and their function [156]. Natural products target the epithelial-mesenchymal transition (EMT), oxidative stress, fibroblast activation, inflammatory injury, metabolic regulation, and extracellular matrix accumulation. The basic mechanisms involved are the NF-*κ*B, TGF-*β*1/Smad, PI3K/Akt, p38 MAPK, Nrf2-Nox4, and AMPK signaling pathways [157]. The plant flavonoid such as eriodictyol was reported to serve as the anti-inflammatory agent in the lungs which regulates the Nrf2 pathway and inhibited the expression of inflammatory cytokines IL-6, TNF-*α*, IL-1*β*, etc. [86]. The flavonoids kaempferol and luteolin reduced the LPS-induced activation of the MAPK and NF-*κ*B pathways and also reported to inhibit the ICAM-1, TNF-*α*, SOD, KC, and neutrophil inflammation. This compound was also found to involve in the reduction of the activity of superoxide dismutase and catalase and further reduces the lipid peroxidation and oxidative damage in the lung tissue [158, 159]. A natural product such as sakuranetin was also reported to reduce the TNF-*α*, eosinophils, M-CSF, RANTES, IL-5, and IL-1*β* and inhibited the NF-*κ*B, MMP-12-positive, and MMP-9-positive cells and also increased the TIMP-1 expression to serve as anti-inflammatory activities in the lungs of the elastase-treated animals [62]. Several compounds such as epigallocatechin, gallocatechin gallate, berberine, berbamine, coptisine, and dicentrine were reported to involve in the inhibition of viral replication, by inhibiting the viral life cycle in the host and act against the viral-induced respiratory inflammations [160]. The 1,8-cineol isolated from the essential oil of *Eucalyptus globulus* leaves was studied for its ability to reduce the expression of NF-*κ*B target gene MUC2 [161]. The 3-methoxy-catalposide had been studied for its ability to inhibit the expression of inducible nitric oxide synthase (iNOS) and cyclooxygenase (COX)-2 in RAW264.7 cells stimulated by LPS. This compound also suppressed the release of nitric oxide (NO) and prostaglandin E2 (PGE2). This compound significantly reduced the activation of inflammatory genes such as interleukins IL-1*β*, IL-6, and TNF-*α* and inhibited the activation of nuclear translocation of NF-*κ*B and AP-1 [162]. Nepitrin, matte flavonoside G, rutin, etc. were reported to inhibit the influenza virus by damaging the viral membrane, by blocking the viral penetration into the cells, and by suppressing neuraminidase in both bacterial and viral infections [163]. Thus, the possible mechanism of action of natural products to reduce the inflammation and diseases in the respiratory system could be by the inhibition of bacteria and viruses and also by the protease-antiprotease balance, NF-*κ*B activation, oxidative stress, and MAPK pathways. The simple flowchart of the mechanism involved is in Figure 3.

## 7. Some Promising Natural Products and Their Pharmacology

Based on the *in vitro* and *in vivo* study, the number of plants based natural products has been studied. Some of them are discussed in detail.

### 7.1. Piperine

Piperine is a major compound and is a class of alkaloid found in the *Piper nigrum* fruits. Piperine was reported to be used in pain management, fever, influenza, hypotension, vascular cell modulation, salivation, stimulation of appetite, antimicrobial, insecticidal, and chills ([164]). This compound was found to enhance the bioavailability of different drugs. Cosupplementation of piperine with resveratrol was reported to increase its efficacy by enhancing bioavailability [165]. Piperine was reported for its dose-dependent activities in reducing the allergic responses, involving sneezing, nasal rubbing, redness of the nose, etc. [166]. This compound was reported to act as an immunomodulatory and antiallergic effect on ova-albumin-induced rhinitis in the rat, by significantly ameliorating the sneezing, coughing, and redness induced by sensitizing. The histopathological section of nasal mucosa showed the attenuation of redness and disruption of alveoli and bronchioles [167]. The antitussive activities of plant extracts containing piperine showed the good enhancement of the antitussive effect [168]. The inhibition of tumor growth in the lungs (B16F-10 melanoma cells) was observed after administration of piperine in the mice. The piperine was found to be 100% cytotoxic to melanoma cells shown by histopathology of lungs, resulted in a significant decrease in tumor mass. The alveolar passage and pleura were tumor-free in the piperine-treated mice [169]. The investigation of the efficacy of curcuminoids co-administered with piperine was measured by measuring the serum level of glutathione (GSH) and malondialdehyde (MDA) in sulfur-mustard-induced chronic pulmonary complications and showed the significant increase in GSH and decrease in MDA indicating improvement in COPD status and health-related quality of life (HRQoL) [170]. There are several other pharmacological activities of piperine that can add to the management of several diseases including respiratory inflammation.

### 7.2. Forsythiaside A

Forsythiaside A is the pharmacologically active monomer of phenylethanoid glycoside. It is the main active ingredient isolated from the fruit and leaves of *Forsythia suspensa.* This compound was reported as a potent component that controls inflammation caused by influenza A virus infection by the molecular mechanism through receptor downregulation of the RLRs signaling pathway. It was reported for anti-inflammatory, antioxidant, and anti-infective activities that explained major biological activities [171]. In a recent study, the anti-inflammatory activity in the lungs of mice had been demonstrated well. Forsythiaside was reported to suppress the inflammatory action of cytokines involving (TNF-*α*, IL-6, and IL-1*β*) via activating Nrf2 and inhibiting the NF-*κ*B signaling pathway in a dose-dependent manner. The number of neutrophils as mediators of inflammation and macrophages was reduced which typically reduced inflammations in the lungs of cigarette and smoke-induced mice [154]. It was reported to act as an immunomodulatory agent which showed an increment in anti-inflammatory cytokines after treatment and restrained the activation of T cell immune response [172]. Forsythiaside A could be developed as a possible therapeutic candidate against respiratory complications.

### 7.3. Mangiferin

Mangiferin, a C-glucosyl xanthone, is a natural polyphenolic compound found in *Mangifera persiciformis*, *Mangifera indica*, *Anemarrhena asphodeloides*, *Salacia hainanensis*, and *Mangifera persiciformis*, along with other plant species [173]. The major source of mangiferin was reported from bark, fruits, roots, and leaves of the papaya tree, peels and kernels of mango fruits, and the leaves, heartwood, and bark of the mango tree [174]. It was reported to reduce the pathological condition that occurred due to inflammation and was effective in inhibiting inflammatory signaling and treating sepsis with acute lung injury (ALI). Mangiferin suppressed respiratory burst and dramatically reduced the expression of NF-*κβ* and proinflammatory cytokines like IL-1, IL-6, and TNF-*α* [175, 176]. An *in vivo* experiment in sepsis-induced mice showed the dose-dependent action of mangiferin upregulated the action of HO-1 (heme oxygenase-1) and mediated the inflammation [177]. Mangiferin had a functional effect on the contraction of tracheal rings. It increased NOS3 protein levels and cGMP levels that prevented muscle contraction in the guinea pig. This preclinical experiment suggested mangiferin to be a potent component for treatment in human lung diseases [178]. It was found to be effective as an immunotherapeutic agent against allergic asthma. The reported results confirmed that mangiferin inhibited PGD2 expression, mediated the level of LTC4, attenuated Th2 cytokines, and displayed a significant role in reducing asthma in a mouse model [179]. The recent studies on mangiferin found the antiallergic properties using a mouse model with allergic rhinitis (AR). The use of mangiferin had a prominent effect in anti-inflammation on nasal tissues. This study further demonstrated the potential of mangiferin in treatment for AR by activating the Nrf2/H-O1 signaling pathway and inhibiting NF-*κ*B [180]. Mangiferin also prevented the formation of the proinflammatory leukotriene LTB4 and decreased the expression of prostaglandin-endoperoxide synthase 2 [173, 181].

### 7.4. Glycyrrhizin

Glycyrrhizin is a triterpene glycoside made up of one molecule of 18-glycyrrhetinic acid and two glucuronic acid molecules of the composition 18-beta-glycyrrhetinic acid-3-O-beta-D-glucuronopyranosyl-(1→2)-beta-D-glucuronide [182, 183]. It is a key active ingredient reported from the root of *Glycyrrhiza glabra* [70]. To examine the effects of glycyrrhizin, a significant anti-inflammatory component found in *G. glabra* was introduced on mice with OVA-induced asthma; it resulted in the alleviation of asthma diseases by lowering the airway hyperreactivity to methacholine, OVA-induced airway constriction, and lung inflammation including significant eosinophil infiltration [70]. Glycyrrhizin was reported for its antiviral properties against a wide range of RNA and DNA viruses. By observing both *in vitro* and *in vivo* experiments, glycyrrhizin had been shown to affect SARS-CoV-2 replication, adsorption, and penetration [184]. Glycyrrhizin dosing could be employed as COVID-19 adjuvant or prophylactic therapy [185]. The data showed that applying glycyrrhizin to the nasal and oral cavities could be the first line of defense against SARS-CoV-2 infection in upper respiratory tract cells. Recent clinical studies of anosmia, hyposmia, and dysgeusia in COVID-19 patients reported the nasal and lingual epithelium serves as a gateway for SARS-CoV-2 entrance [186, 187]. This hypothesis is supported by the fact that glycyrrhizin possesses excellent physical features such as amphiphilicity and the capacity to change the characteristics of lipid bilayer membranes.

### 7.5. Curcumin

Curcumin is a polyphenolic compound that is biologically active and found in the roots of *Curcuma longa.* It is the active component having wide pharmacological benefits. This compound was reported to suppress inflammation and showed pulmonoprotective effects. It inhibited the NF-*κ*B and mitogen-activated protein kinase (MAPK) signaling pathways. Treatment with curcumin attenuated the secretion of TNF-*α*, IFN-*α*, and IL-6 and deals efficiently with the complications [188]. The efficacy of curcumin was reported by various pieces of evidence in lung diseases and was found to be effective and reliable to be used in various respiratory complications like asthma, COPD, lung cancer, and other lung injuries. It was reported to reduce the degree of inflammatory cells and alleviates dysregulation [189]. Curcumin was reported to hold the ability to bind with receptors, blocked the entry of the virus into the cells, and interfered with its replication. Lung inflammation due to COVID-19 can be mediated by its uses. Some reports from *in silico* analysis supported the issue. This potential serves to recommend its implication in therapeutics in COVID-19-induced respiratory complications [190].

### 7.6. Zingerone

Zingerone is the major component found in the ginger root to about 9.25%. This compound was reported to be closely related to the vanillin from vanilla and eugenol from clove [191]. This compound was reported as a nontoxic compound bearing various pharmacological importance. This compound was extensively studied for its effect on lung injuries. It significantly lessened the pulmonary edema, attenuated the amount of TNF-*α* and IL-*β* in BALF, and inhibited proinflammatory cytokine release in acute lung injury in mice [151]. The hepatoprotective effect of zingerone had been studied in the LPS-induced hepatic injury in mice in terms of liver histology, liver function marker, and several other inflammatory markers such as TNF-*α*, TLR4, and iNOS parameters. The zingerone-treated group showed significant improvement in liver histology, decreased endotoxin level, improved liver function markers, and downregulation of mRNA expression of TNF-*α*, TLR4, and iNOS indicating better anti-inflammatory activities.

### 7.7. Vitexin

Vitexin (apigenin-8-C-*β*-D-glucopyranoside) is a flavone glycoside of apigenin found in food and medicinal plants such as the hawthorn leaf [192], bamboo [193], buckwheat [194], Passiflora [195], and Echinodorus [196]. Vitexin was reported as a significant polyphenol present in foods such as mung beans [197], which are frequently utilized in traditional Chinese medicine [192]. In the gastrointestinal tract, vitexin is poorly absorbed. It is rapidly eliminated from the bloodstream, primarily eliminated in the urine and bile [198]. This compound is reported to have very poor absolute oral bioavailability and is quickly and broadly disseminated throughout the body. The buildup of reactive oxygen species (ROS) exacerbated inflammatory reactions by boosting the release of proinflammatory cytokines and inflammatory cell infiltration [199]. When compared to vehicle-treated mice, vitexin administration reduced LPS-induced ROS levels by 44%. Vitexin therapy reduced neutrophils and the production of proinflammatory cytokines. This compound reduced pulmonary edema and protein concentration in the alveoli. The activity of Nrf2 and HO-1 was significantly increased after treatment with vitexin. Vitexin also boosted the activity of its target gene, heme oxygenase (HO)-1, via activating nuclear factor erythroid-2-related factor 2 (Nrf2) [103].

## 8. Conclusion and Future Perspective

In this review, the drawbacks and limitations of currently adopted treatment procedures and available drugs have been highlighted. This study also reported the several plant species that are being used in the treatment of respiratory complications in the traditional medicinal system based on traditional knowledge and indigenous knowledge. The reported bioactive compounds and their mechanism of action have been critically analyzed for possible therapeutic compounds. Some of the plant products are promising against respiratory diseases and can be the best source of alternative medicine. Although, some clinical shreds of evidence have been reported for some of the compounds, there needs to be an extensive study on the toxicological aspect and interaction with other therapeutics. The detail studies on the formulations, forms of doses, evaluation of pharmacokinetic parameter, and safety are necessary. The future study should focus on the identification and isolation of more effective compounds, their mechanism of action, and formulations. This study can facilitate the newly discovered compounds to enter a clinical trial. Therefore, it is concluded that further research on the traditionally used plants and plant-derived products could lead to the discovery of a new kind of therapeutic drug of high potential and interest.

## Figures and Tables

**Table 1:** Traditionally used plants in different countries and localities against respiratory disorder.

S.N.	Plant names (local name if available)	Country (locality if available)	Plant parts used	Forms	Mode of application	Traditional use	References
1.	*Abies pindrow*	Pakistan (Kashmir)	Bark	Powder	Internal	Cough, chronic asthma	[40]
2.	*Abies pindrow* (partal)	Pakistan (Kashmir)	Root	Decoction	Internal	Cough, bronchitis	[40]
3.	*Abrus precatorius* (omisinmisin)	Nigeria (Osun State)	Leaves	Decoction	Oral	Asthma bronchitis, cough, tuberculosis	[42]
4.	*Acalypha indica*	Myanmar (Mon)	Whole plant	Juice	Oral	Asthma	[43]
5.	*Acanthus pubescens* (Amatojo)	Uganda	Root	Boiled	Oral	Cough	[44]
6.	*Achyranthes aspera* (Puthkanda)	Pakistan(Gujranwala)	Leaves	Decoction	Oral	Pneumonia	[45]
7.	*Achyranthes aspera* (Puthkanda)	Pakistan (Soan Valley)	Root	Decoction, juice	Oral	Pneumonia	[46]
8.	*Aconitum ferox* (Seto bikhma)	Nepal	Root	Dried root juice	Oral	Cough	[47]
9.	*Aconitum heterophyllum*	Nepal (Rasuwa)	Root	Powder	Oral	Cough	[48]
10.	*Aconitum heterophyllum*	Pakistan (Dawarian Village)	Root	Boiled	Internal	Flu cough	[49]
11.	*Acorus calamus* (Bojho)	Nepal	Root	Juice small piece	Orally	Bronchitis, to clear the throat and open the voice	[41]
12.	*Adiantum capillus-veneris* (Hansraj, Sraj fern)	Pakistan (Kashmir)	Leaves	Decoction	External	Cough, asthma	[40]
13.	*Adiantum capillus-veneris* (Khati booti)	Pakistan (Soan Valley, Salt Range)	Whole parts	Tea	Oral	Coughs, bronchitis, and pneumonia	[46]
14.	*Adiantum capillus-veneris*	Pakistan (Dawarian Village)	Fruit	Raw fruit	Internal	Cough	[49]
15.	*Aegle marmelos* (Bilpatre, Bael)	India (Shimoga)	Leaves	Boiled	Oral	Asthma	[18]
16.	*Aerva javanica var. javanica* (Boo)	Pakistan	Inflorescence	Decoction	Internal	Asthma	[17]
17.	*Albizia lebbeck Benth* (Sharin)	Pakistan (Gujranwala)	Flowers	Decoction	Oral	Asthma	[45]
18.	*Alhagi maurorum* (Puthkanda)	Pakistan (Punjab)	Whole parts	Decoction, juice, infusion	Oral	Asthma	[50]
19.	*Alhagi maurorum* (Puthkanda)	Pakistan (Punjab)	Flowers, leaves, seed, fruit, and stem	Decoction, juice, infusion, powder, vegetable, paste, poultice, and tea	Topical, oral	Asthma, cough	[51]
20.	*Allium cepa*	Pakistan (Punjab)	Stem, leaves	Decoction, infusion, paste, and juice	Oral	Cough	[51]
21.	*Allium cepa*	Pakistan (Punjab)	Stem, leaves	Decoction, juice	Internal	Cough	[50]
22.	*Allium fasciculatum* (Rendle Faran, Farun, Chyapi)	Nepal (Rasuwa)	Whole plant	Paste	Oral	Sore throat	[48]
23.	*Allium hypsitum* (Sternb. Chyapi, Ban Lasun, Jimbu, Jimbu jhar)	Nepal (Rasuwa)	Whole plant	Powder	Oral	Cough	[48]
24.	*Allium sativum* (Lahsan)	Pakistan (Punjab)	Stem, leaves	Decoction, infusion, paste, tea, and juice	Oral	Asthma	[51]
25.	*Allium sativum* (Thoom)	Pakistan (Gujranwala)	Bulb	Juice	Oral	Respiratory tract infection	[45]
26.	*Alysicarpus vaginalis*	Myanmar (Mon)	Whole plant	Juice	Oral	Asthma and cough	[43]
27.	*Amaranthus albus* (Soor, Booti)	Pakistan (Punjab)	Flowers, leaves, seed, and stem	Decoction, juice, infusion, and poultice	Topical	Asthma	[51]
28.	*Amaranthus albus* (Soor Booti)	Pakistan (Punjab)	Whole plant	Decoction, juice	Internal	Pneumonia	[50]
29.	*Anethum graveolens*	Pakistan (Kashmir)	Fruit	Powdered	Internal	Cough and asthma	[49]
30.	*Annas cosmas*	—	Fruit	Raw plant	Oral	Asthma	[52]
31.	*Artemisia indica* (Tite pati)	Nepal	Leaves	Juice	Oral	Bronchitis	[41]
32.	*Asphodelus tenuifolius Cav* (Piyazi)	Pakistan (Punjab)	Leaves, stem	Juice, infusion, powder	Internal	Cough	[50]
33.	*Astilbe rivularis* (Thulo ausadhi)	Nepal (Rasuwa)	Root	Powder	Oral	Cough	[53]
34.	*Avena sativa* (Jai)	Pakistan (Punjab)	Whole parts	Powdered	Oral	Cough	[50]
35.	*Averrhoa carambola*	Nepal	Fruit	Powder, boil with water or milk	Oral	Against COVID-19 virus	[54]
36.	*Azadirachta indica* (Niimu)	Uganda	Leaves	Juice	Oral	Cough	[44]
37.	*Bergenia ciliata* (Sternb. Pakhanbed)	Nepal	Rhizome	Root powder	Oral	Cough, tonsillitis	[47]
38.	*Bergenia ciliate Haw.* (Zakhm-e-Hayat)	Pakistan (Kashmir)	Root	Juice	Internal	Cough and cold	[40]
39.	*Bistorta amplexicaulis* (Masloon)	Pakistan	Leaves and roots	Powder	Oral	Respiratory disorders	[55]
40.	*Bombax ceiba* (Simal)	Nepal	Root	Decoction	Oral	Bronchitis	[41]
41.	*Bothriocline longipes* (Ekyoganyanja)	Uganda	Leaves	Juice	Oral	Cough	[44]
42.	*Callistemon citrinus* (Curtis, Skeels)	Uganda	Leaves	Boiled juice	Oral	Cough	[44]
43.	*Calotropis gigantea*	India	Root and leaves	Decoction	Oral	Shortness of breath	[56]
44.	*Calotropis gigantea* (Aank)	Nepal	Root, milky latex, and flowers	Paste	Oral	Cough, bronchitis	[41]
45.	*Capparis zeylanica* (Kurutigana, Soppu)	India (Shimoga)	Leaves	Juice	Oral	Cough	[18]
46.	*Cardia myxa* (Lasoora)	Pakistan (Punjab)	Fruit, stem, leaves, and bark	Decoction, juice, vegetable, infusion, and powder	Oral	Respiratory tract infection	[51]
47.	*Cardiospermum halicacabum* (Bekkina Budde gida)	India (Shimoga)	Leaves	Smoke	Inhale	Cough	[18]
48.	*Carica papaya*	—	Leaves	Juice	Oral	Asthma	[52]
49.	*Carissa carandas* (Kavali)	India (Shimoga)	Root	Juice	Oral	Asthma	[18]
50.	*Carum carvi* (Bhote jeera, Sim jeera)	Nepal (Rasuwa)	Fruit, whole plant	Fruits	Oral	Cough	[48]
51.	*Cassiope fastigiata* (Maudhupi)	Nepal (Rasuwa)	Leaves	Leaves infusion	Oral	Cough	[48]
52.	*Castanea sativa* (chestnut)	Pakistan (Kashmir)	Leaves	Decoction	Internal	Sore throat	[40]
53.	*Centella asiatica* (Kutukumwe)	Uganda	Leaves	Juice	Oral	Cough	[44]
54.	*Chenopodium album* (Lullar)	Pakistan	Fruit	Powdered	Internal	Asthma, whooping cough	[17]
55.	*Chromolaena odorata*	Myanmar (Mon)	Whole plant	Juice	Oral	Cough	[43]
56.	*Chrysanthemum indicum* (Gul-e-Daudi)	Pakistan (Punjab)	Flowers, leaves, and stem	Decoction, juice, and powdered	Internal	Cough	[50]
57.	*Clematis gouriana* (Ballivadaka, Gourian clematis)	India (Shimoga)	Flowers	Powder	Oral	Asthma	[18]
58.	*Clematis montana* (Langi)	Pakistan (Kashmir)	Flowers	Decocted	Internal	Cough	[40]
59.	*Coccinia grandis* (Voigt Golkakri)	Nepal	Root	Root extract	Oral	Pneumonia, tonsillitis, and throat infection	[47]
60.	*Conyza bonariensis* (Choozni)	Pakistan (Punjab)	Leaves, stem	Powdered, juice, and infusion	Internal	Cough	[50]
61.	*Coriandrum sativum* (Dhaniya)	Pakistan (Punjab)	Whole parts	Decoction and vegetable	Oral	Respiratory tract infection	[51]
62.	Coriandrum sativum	Pakistan (Chitral)	Fresh leaves and dried fruits	Decoction	Internal	Bronchitis	[57]
63.	*Cressa cretica* (Bukkan)	Pakistan	Whole plant	Decoction	Internal	Asthma	[17]
64.	*Cymbopogon citratis* (Pire ghans)	Nepal	Leaves	Tea	Oral	Cough	[41]
65.	*Cymbopogon jwarancusa* (Nadak)	Pakistan	Whole plant	Decoction	Internal	Cough, bronchitis	[17]
66.	*Delphinium himalayae* (Bhongmar)	Nepal (Rasuwa)	Root	Extract	Oral	Cough	[53]
67.	*Dicarnopteris linearis* (Muikandochla)	India (Tripura)	Fronds	Decoction	Oral	Throat pain	[58]
68.	*Dicliptera bupleuroides* (Kirch, somni)	Pakistan (Kashmir)	Leaves	Decoction	External	Treatment of cough	[40]
69.	*Elaeagnus angustifolia*	Pakistan (Kashmir)	Fruit	Raw fruit	Internal	Cough and cold	[40]
70.	*Elaeagnus angustifolia*	Pakistan (Kashmir)	Ripe fruits	Boiled	Internal	Sore throat	[40]
71.	*Elaeagnus umbellate* (Russian Olive)	Pakistan (Kashmir)	Leaves	Decoction	Internal	Cough	[40]
72.	*Embelia ribes* (Vayuvilanga)	India (Shimoga)	Root	Juice	Oral	Cough	[18]
73.	*Enicostemma hyssopifolium*	Pakistan	Whole plant	Decoction	Internal	Cough	[17]
74.	*Eucalyptus grandis* (Karutusi)	Uganda	Leaves	Boiled Juice	Oral	Cough	[44]
75.	*Euphorbia heliscopia* (Dhodak)	Pakistan (Punjab)	Whole parts	Decoction, juice, infusion	Oral	Cough	[50]
76.	*Euphorbia hirta* (Kippo)	Pakistan	Whole plant	Decoction	Internal	Asthma	[17]
77.	*Euphorbia hirta* (Dudhi jhar)	Nepal	Leaves	Dried/soaked	Oral	Against COVID-19 virus	[54]
78.	*Euphorbia hirta* (Khemychu)	India (Tripura)	Leaves	Juice	Gargling	Throat pain	[58]
79.	*Euphorbia prostate* (Dhodak)	Pakistan (Punjab)	Whole parts	Decoction, juice, infusion	Oral	Cough	[50]
80.	*Gentiana kurroo Royle* (Spanthing)	Pakistan (Baltistan)	Flowers	Infusion	Oral	Cough	[59]
81.	*Gentianodes tianschanica*	Pakistan (Baltistan)	Leaves	Infusion	Oral	Pneumonia, bronchitis, cough	[59]
82.	*Glycyrrhiza glabra* (Jhestamadhu)	India (Shimoga)	Root	Powder	Oral	Asthma	[18]
83.	*Helianthus annus* (Suraj Makhi)	Pakistan (Punjab)	Whole plant	Powder, paste, ash	Internal	Respiratory tract infection	[50]
84.	*Helichrysum schimperi* (Moeser, Ekyeeza)	Uganda	Leaves	Powder	Oral	Pneumonia	[44]
85.	*Hydrocotyl verdicillta*	Myanmar (Mon)	Whole plant	Decoction	Oral	Asthma	[43]
86.	*Justica adhatoda* (Vahaekar)	Pakistan (Soan Valley)	Leaves and roots	Juice	Oral with ginger	Cough	[46]
87.	*Justicia adhatoda* (Baykr)	Pakistan (Gujranwala)	Leaves and flowers	Decoction	Oral	Cough	[45]
88.	*Justicia adhatoda* (Asuamfang)	India (Tripura)	Root, leaves	Decoction, juice	Oral	Pneumonia and cough	[58]
89.	*Justicia adhatoda*	India (Shimoga)	Root	Root paste with human breast milk	Oral	Bronchitis	[18]
90.	*Lantana camara* (Lantani)	Pakistan (Punjab)	Leaves, stem	Juice, infusion, powder	Internal (oral)	Asthma	[50]
91.	*Malva parviflora* (Sunchal)	Pakistan (Gujranwala)	Leaves	Decoction	Oral	Cough	[45]
92.	*Malva parviflora* (Ekituruguma)	Uganda	Leaves	Powder	Oral	Pneumonia	[44]
93.	*Mangifera indica* (L. Omuyembe)	Uganda	Bark	Boiled	Oral	Cough	[44]
94.	*Mentha riyleana* (Podina)	Pakistan (Kashmir)	Leaves	Juice	Internal	Cough	[40]
95.	*Mentha spicata* (Podina)	Pakistan (Sudhanoti)	Leaves, root	Paste	Oral	Cough, throat pain	[60]
96.	*Mimosa pudica* (Hta Muck)	Myanmar (Mon)	Whole plant	Juice	Oral	Cough	[43]
97.	*Mondia whitei* (Hook. F, Skeels, Omurondo)	Uganda	Root	Powder	Chew orally	Cough	[44]
98.	*Morus alba*	Pakistan (Dawarian Village)	Leaves	Boiled	Internal	Sore throat	[49]
99.	*Morus alba* (Cheeta Toot)	Pakistan (Sudhanoti)	Flowers, root	Paste	Oral brush	Cough	[60]
100.	Morus alba	Korea	Root bark	Paste	Oral	Cough, bronchitis, and asthma	[39]
101.	*Morus nigra*	Pakistan (Dawarian Village)	Fruit pulp	Syrup	Internal	Sore throat	[49]
102.	*Morus nigra* (Kala too)	Pakistan (Gujranwala)	Fruit	Juice	Oral	Sore throat, cough	[45]
103.	*Nepeta erecta* (Boyle ex Benth Berth Mominan)	Pakistan (Baltistan)	Leaves	Infusion	Oral	Cough	[59]
104.	*Nepeta erecta Royle ex.*	Pakistan (Kashmir)	Flowers	Juice	Internal	Cough	[40]
105.	*Ocimum suave* Wild., (Omujaaja)	Uganda	Leaves	Boiled Juice	Oral	Cough	[44]
106.	*Ocimum tenuiflorum* (Krishna Tulsi)	Nepal	Whole plant	Decoction	Oral	Cough	[41]
107.	*Onosma bracteatum Wall*	Pakistan (Dawarian Village)	Root	Powdered	Internal	Asthma and bronchitis	[49]
108.	*Otthochloa compressa* (Nooli)	Pakistan (Punjab)	Leaves, stem	Decoction, juice, tea	Oral	Cough	[50]
109.	*Oxalis debilis* (Teenpatra)	Pakistan	Leaves	Powder	Oral	Asthma	[55]
110.	*Paris polyphylla* Sm. (Satuwa)	Nepal	Root	Powder juice	Oral	Cough	[47]
111.	*Persea Americana* (Ovacado)	Uganda	Leaves	Boiled juice	Oral	Cough	[44]
112.	*Phalaris minor* (Dumbi sitt)	Pakistan (Punjab)	Stem, leaves	Infusion, paste	Oral	Cough	[51]
113.	*Phyllanthus emblica* (Amala)	Nepal	Bark and fruit	Juice	Oral	Shore throat	[41]
114.	*Piper longum* (Pipla)	Nepal	Fruit	Fruits	Oral	Cough	[47]
115.	*Pisum sativum* (Mattr)	Pakistan (Punjab)	Whole parts	Decoction, juice, infusion	Oral	Asthma	[50]
116.	*Plantago palmata* (Embatabata)	Uganda	Leaves	Powder	Oral	Pneumonia	[44]
117.	*Plectranthus barbatus* (Ekicuncu)	Uganda	Leaves	Juice	Oral	Cough	[44]
118.	*Populus tremula* (Peepal)	Pakistan (Punjab)	Leaves, bark	Decoction, juice, and infusion	Oral	Cough	[51]
119.	*Portulaca quadrifida* (Dasi kulfa)	Pakistan (Gujranwala)	Leaves	Infusion	Oral	Respiratory problems	[45]
120.	*Prunus persica Linn* (Aru)	Pakistan (Kashmir)	Leaves	Juice	Internal	Cough, bronchitis	. [40]
121.	*Punica granatum* (Druna)	Pakistan (Kashmir)	Fruit	Raw fruit	Internal	Cough	[40]
122.	*Punica granatum*	Pakistan (Chitral)	Fruit rind	Raw fruit	Internal	Whooping cough	[57]
123.	*Quercus baloot* (Rein, Shah Baloot, Oak)	Pakistan (Kashmir)	Bark	Powder	Internal	Asthma	[40]
124.	*Quercus incana* (Rein, Ban, Rinji)	Pakistan (Kashmir)	Bark	Powder	Internal	Asthma, cough	[40]
125.	*Ranunculus muricatus*	Pakistan (Kashmir)	Aerial parts	Cooked	Internal	Asthma	[40]
126.	*Rheum acuminatum* (Thomson Khokim)	Nepal	Rhizome	Rhizome	Oral	Cough	[47]
127.	*Rhodiola imbricata* (Edgew Chundol)	Pakistan (Baltistan)	Root	Powder	Oral	Cough	[59]
128.	*Rhoicissus tridentata* (Drumm., Omumara)	Uganda	Leaves	Boiled juice	Oral	Cough	[44]
129.	*Rhus vulgaris Meikle* (Omukanja)	Uganda	Fruit	Raw fruit	Oral	Cough	[44]
130.	*Rubia cordifolia* (Akaramata)	Uganda	Leaves	Juice	Oral	Pneumonia	[44]
131.	*Rumex chalepensis* (Khar palak)	Pakistan (Punjab)	Leaves, stem	Decoction, juice, tea	Oral	Cough	[50]
132.	*Rumex dentatus* (Khar palak)	Pakistan (Punjab)	Leaves, stem	Decoction, juice, tea	Oral	Cough	[50]
133.	*Rumex hastatus* (Khatimal)	Pakistan (Kashmir)	Root	Juice	Internal	Cough, asthma	[40]
134.	*Saccharum officinarum*	Nigeria (Osun State)	Stem	Maceration	Oral	Asthma, respiratory diseases in children	[42]
135.	*Salsola baryosma* (Khaar)	Pakistan (Punjab)	Stem, leaves	Decoction, infusion, and juice	Oral	Cough	[51]
136.	*Salvia hians*	Pakistan (Kashmir)	Leaves	Juice	Internal	Cough	[40]
137.	*Senegalia rugata* Lam.	Myanmar (Mon)	Leaves/whole plant	Decoction	Oral	Asthma	[43]
138.	*Solanecio cydoniifolius* (Eirarira)	Uganda	Root	Boiled	Oral	Cough	[44]
139.	*Solanum surattense* (Mookri)	Pakistan (Gujranwala)	Root	Tea	Oral	Asthma	[45]
140.	*Solanum surratense* (Kndyari)	Pakistan (Punjab)	Whole parts	Decoction, juice, infusion, paste, tea	Internal	Respiratory tract infection	[50]
141.	*Sonchus wightianus* (Mulapate)	Nepal	Root	Raw root	Oral	Tonsillitis	[47]
142.	*Spinacia oleracea* (Palaki)	Pakistan (Punjab)	Leaves	Decoction, juice	Internal	Cough	[50]
143.	*Swertia chirayita* (Karsten Chiraito)	Nepal	Whole plant	Boiled juice	Oral	Cough	[47]
144.	*Swertia ciliate G*	Pakistan (Kashmir)	Aerial part	Decoction	Internal	Cough	[40]
145.	*Swertia cordata* (G. Don, Clarke Karfo sman)	Pakistan (Baltistan)	Flower	Powder	Oral with water	Cough	[59]
146.	*Tagetes erecta* (Gainda)	Pakistan (Punjab)	Flowers, leaves, and fruit	Decoction, juice, infusion, and powder	Oral	Asthma, respiratory tract infection	[51]
147.	*Tagetes erecta* (Gaindi)	Pakistan (Punjab)	Stem, leaves, and flowers	Decoction, powder, paste	Topical	Asthma	[50]
148.	*Taverniera persica*	Pakistan (Punjab)	Fruit, stem, leaves, and seed	Decoction, infusion, powder, vegetable, and poultice	Oral	Cough	[51]
149.	*Terminalia bellirica* (Barro)	Nepal	Stem bark and fruit	Juice	Oral	Cough	[41]
150.	*Terminalia chebula* (Harro)	Nepal	Fruit	Juice	Oral	Cough	[41]
151.	*Tetradenia riparia* (Omuravunga)	Uganda	Leaves	Boiled juice	Oral	Cough	[44]
152.	*Tinospora sinensis* (Sin-don-manwe)	Myanmar (Mon)	Root/stem	Decoction	Oral	Cough	[43]
153.	*Trianthema portulacastrum*	Pakistan	Leaves	Decoction	Internal	Asthma	[17]
154.	*Trianthema portulacastrum*	Pakistan (Gujranwala)	Root	Decoction	Oral	Asthma	[45]
155.	*Trianthema triquetra Rottl.* (Chulani)	Pakistan (Punjab)	Flowers, leaves, and stem	Decoction, tea	Oral	Cough	[51]
156.	*Trichodesma indicum* (Handusi booti)	Pakistan (Jammu and Kashmir)	Leaves	Boiling	Internal	Cough	[40]
157.	*Trifolium alexandrium* (Berseem)	Pakistan (Punjab)	Stem, leaves	Decoction, juice, vegetable, and paste	Oral	Respiratory tract infection	[51]
158.	*Tussilago farfara* (Churut)	Pakistan (Baltistan)	Leaves	Infusion	Oral	Cough, respiratory problems	[59]
159.	*Valeriana hardwickii* Wall. (Samayo, Nakali Jatamansi)	Nepal (Rasuwa)	Root	Root paste	Oral	Cough	[48]
160.	*Valeriana jatamansii* (Jones Samayo, Sugandhawal)	Nepal (Rasuwa)	Root	Root paste	Oral	Cough	[48]
161.	*Verbascum thapsus* (Guni puchar)	Nepal (Rasuwa)	Timber	Root paste	Oral	Asthma	[48]
162.	*Vernonia amygdalina*	Nigeria (Osun State)	Leaves, stem, bark	Maceration	Oral	Asthma, cough, tuberculosis	[42]
163.	*Viburnum grandifloum* (Guch)	Pakistan (Kashmir)	Seed	Juice	Internal	Whooping cough	[40]
164.	*Viola canescens* (Pholala)	Pakistan	Leaves and roots	Paste	Oral	Cough and respiratory problems	[55]
165.	*Viola canescens Ex* (Banafsa)	Pakistan (Kashmir)	Root	Juice	Internal	Cough and cold	[40]
166.	*Viola canescens*	Pakistan (Dawarian Village)	Leaves and flowers	Decoction	Internal	Bronchitis, respiratory catarrh, coughs, and asthma	[49]
167.	*Viola odorata* (Banafshaa)	Pakistan (Sudhanoti)	Flowers, leaves, root	Paste	Oral, topical	Cough, cure throat infection	[60]
168.	*Vitex negundo* (Simali)	Nepal	Leaf juice	Juice	Oral	Cough	[41]
169.	*Vitex trifolia* (Kyaung-ban-lay)	Myanmar (Mon)	Bark	Decoction	Oral	Cough/asthma	[43]
170.	*Vitis vinifera*	Pakistan (Dawarian Village)	Flowers	Decoction	Internal		[49]
171.	*Zanthoxylum armatum* (Timur)	Nepal	Fruit	Fruit	Oral	Cough	[47]
172.	*Zingiber officinale* (Roscoe)	Uganda	Stem	Juice	Oral	Cough	[44]
173.	*Zingiber officinale* (Aduwa)	Nepal	Rhizome	Juice	Oral	Cough	[41]

**Table 2:** Plant-derived compounds associated with respiratory inflammation.

S.N.	Constituents	Plant origin	Doses	Inflammagen used	References
Alkaloids					
1.	Warifteine	*Cissampelos sympodialis*	2 mg/kg	OVA-induced	[74]
2.	Colchicine	*Colchicum autumnale*	0.25-0.5 mg/kg	Idiopathetic pulmonary fibrosis	[73]
3.	Imperialine	*Fritillaria cirrhosa*	3.5-7 mg/kg	Cigarette smoke or LPS	[75]
4.	Piperine	*Piper longum*	2.25-4.5 mg/kg	Ovalbumin	[67]
5.	Cepharanthine	*Stephania cepharantha*	5 mg/kg	LPS	[76]
6.	Nimbandiol	*Azadirachta indica*	(*in silico*)	—	[77]
7.	Vasicine	*Peganum harmala*	45 mg/kg	Ammonia liquor, capsaicin, and citric acid	[78]
8.	Vasicinone				
9.	Deoxyvasicine				

Cannabinoids					
10.	Cannabidiol	*Cannabis sativa*	20 mg/kg	LPS	[79, 80]

Flavonoids					
11.	Pinocembrin (5,7-dihydroxyflavanone)	*Alpinia katsumadai*	20-50 mg/kg	LPS	[81]
12.	Naringenin	*Prunus persica*	15-100 mg/kg	LPS, *Staphylococcus aureus*	[82, 83]
13.	Naringenin	*Vitis vinifera*	100-200 mg/kg	Radiations (*γ*-ray)	[84]
14.	Alpinetin	*Alpinia katsumadai*	50 mg/kg	LPS	[85]
15.	Eriodictyol	*Dracocephalum rupestre*	30 mg/kg	LPS	[86]
16.	Licorice flavonoid (liquiritin)	*Glycyrrhiza uralensis*	30 mg/kg	LPS	[87]
17.	Isoliquiritigenin (ILG)	*Glycyrrhiza glabra*	10-30 mg/kg	Cigarette smoke	[88]
18.	Baicalin	*Scutellaria baicalensis*	20-80 mg/kg	Cigarette smoke-induced (rat model)/ovalbumin (OVA)/influenza H1N1	[89–91]
19.	Oroxylin A				
20.	Wogonin				
21.	Chrysin				
22.	Moracins	*Morus alba*	20-60 mg/kg	LPS	[92]
23.	Sakuranetin	*Baccharis retusa*	20 mg/kg	Elastase-induced emphysema	[62]
24.	Schaftoside	*Eleusine indica*	0.4 mg/kg	LPS	[93]
25.	Kuwanone E	*Morus alba*	200–400 mg/kg	LPS	[61]
26.	Kuwanone G				
27.	Norartocarpanone				
28.	Luteolin	*Mosla chinensis*	288-576 mg/kg	LPS	[94]
29.	Mosla scabra flavonoids	*Mosla scabra*	30-90 mg/kg	LPS	[95–97]
30.	Apigenin	*Allium cepa*, *Citrus X sinensis*	10-20 mg/kg	LPS	[98]
31.	Myricetin	*Abelmoschus moschatus*	100 mg/kg	Bleomycin	[99]
32.	Icariin	*Epimedium brevicornu*	—	Ova-induced	[100]
33.	Fisetin	*Cucurbita pepo*	1-3 mg/kg	Ova-induced	[101, 102]

Glycosides					
34.	Vitexin	Leaf of *Crataegus*	10 mg/kg	LPS	[103]
35.	Hyperin	*Houttuynia cordata*	50-200 mg/kg	Influenza virus H1N1	[104]
36.	Quercitrin	*Houttuynia cordata*	100 mg/kg	LPS/influenza virus H1N1	[104, 105]
37.	Picroside II	*Picrorhiza scrophulariiflora*	0.5-1 mg/kg	LPS	[106]

Lignans					
38.	Magnolol	*Magnolia officinalis*	5-20 mg/kg	LPS	[107]
39.	Phillyrin	*Forsythia suspensa*	10-20 mg/kg	LPS	[108]
40.	Columbianadin	*Angelica decursiva*	20–60 mg/kg	LPS	[68]
41.	Schisantherin A	*Schisandra sphenanthera*	40 mg/kg	LPS	[109]
42.	Schisantherin B	*Schisandra chinensis*	15-60 mg/kg	OVA-induced	[110]

Macromolecular polymer					
43.	Lipopolysaccharides	*Houttuynia cordata*	40-160 mg/kg	LPS	[111]
44.	Polysaccharides	*Houttuynia cordata*	20-40 mg/kg	Influenza A virus (IAV) H1N1	[112]

Polyphenols					
45.	Resveratrol		50 mg/kg	OVA-induced allergy	[65, 113]
46.	Luteolin	*Lonicera japonica*	18–70 *μ*mol/kg	LPS	[66]
47.	Curcumin	*Curcuma longa*	150 mg/kg	*Klebsiella pneumoniae*	[63, 64]

Saponins					
48.	Lugrandoside	*Digitalis lutea* and *Digitalis grandiflora*	10-30 mg/kg	LPS	[114]
49.	Ginsenosides	*Panax ginseng*	20 mg/kg	LPS	[115]
50.	Methyl protodioscin	*Asparagus cochinchinensis*	30-60 mg/kg	LPS	[116]
51.	Glycyrrhizin	*Glycyrrhiza glabra*	2.5-20 mg/kg	OVA-induced allergy	[69, 70]
52.	Mogroside V	*Momordica grosvenori*	2.5-10 mg/kg	LPS	[117]
53.	Hederacoside C	*Hedera helix*	50 mg/kg	*S. aureus*	[118, 119]
54.	Platycodin D	*Platycodon grandiflorum*	50-100 mg/kg	LPS	[120]
55.	Rhodiocyanoside A	*Rhodiola rosea*	200-800 mg/kg	Cigratte smoke and LPS	[121]
56.	Stevioside	*Stevia rebaudiana*	12.5-50 mg/kg	LPS	[122]
57.	Hesperidine	*Mentha piperita*	(*in silico*)	—	[123]

Terpenoids					
58.	Patchouli alcohol	*Pogostemon cablin*	10-40 mg/kg	LPS	[124]
59.	Pogostone	*Pogostemon cablin*	10-40 mg/kg	LPS	[125]
60.	Andrographolide	*Andrographis paniculata*	0.1-1 mg/kg	Cigarette smoke (CS)	[126–128]
61.	Geraniol	*Citrus X lemon*, *rosa*, *Zingiber officinale Rosc.*, and *Citrus X sinensis*	12.5-50 mg/kg	LPS	[129, 130]
62.	Carvacrol	*Plectranthus amboinicus*, *Zataria multiflora*	20-80 mg/kg	LPS	[131, 132]
63.	Isoforskolin	*Coleus forskohlii*	5-20 mg/kg	LPS	[133]
64.	Sclareol	*Salvia sclarea*	2.5-10 mg/kg	LPS	[134]
65.	Triptolide	*Tripterygium wilfordii*	5-15 *μ*g/kg	LPS	[135]
66.	Thymoquinone	*Nigella sativa*	5-10 mg/kg	LPS	[136]
67.	Oridonin	*Rabdosia rubescens*	20-40 mg/kg	LPS	[137]
68.	*β*-Patchoulene	*Pogostemon cablin*	10 mg/kg	LPS	[138]
69.	Taraxasterol	*Taraxacum officinale*	2.5-10 mg/kg	LPS	[139]
70.	1,8-Cineol	*Eucalyptus globulus*	10^−4^ M	LPS	[113]
71.	Fridelin	*Euphorbia nerifolia*	5 *μ*g/mL	COVID-19	[140]
72.	Asiatic acid	*Centellae asiaticae herba*	25-100 mg/kg	LPS	[141, 142]

Others					
73.	Mangiferin	*Mangifera indica*	0.45-4.5 mg/kg	LPS	[143]
74.	Ergosterol	*Scleroderma polyrhizum*	25-50 mg/kg	LPS	[144]
75.	Crytotanshinone	*Salvia miltiorrhiza*	10-40 mg/kg	LPS	[145]
76.	Prime-O-glucosylcimifugin	*Saposhnikovia divaricata*	2.5-10 mg/kg	LPS	[146]
77.	Usnic acid	*Lichen* spp.	50-100 mg/kg	LPS	[147]
78.	Shikonin	*Lithospermum erythrorhizon*	12.5-50 mg/kg	LPS	[148, 149]
79.	Linalool	Aromatic plant	10-40 mg/kg	Cigarette smoke	[150]
80.	Zingerone	*Zingiber officinale*	10-40 mg/kg	LPS	[151]
81.	Paeonol	*Paeonia suffruticosa*	10 mg/day	Cigarette smoke (CS)	[152]
82.	Acteoside	*Rehmannia glutinosa*	30-60 mg/kg	LPS	[153]
83.	Forsythiaside A	*Forsythia suspensa*	15-60 mg/kg	Cigarette smoke	[154]
84.	Chloroform	*Pyrossia lingua*	2378 *μ*g/mL	COVID-19	[140]
85.	3,4-Di-O-caffeoylquinic acid	*Lonicera japonica*	68.3 *μ*M	Virus	[155]
